# Development and validation of a nomogram to predict depression in older adults with heart disease: a national survey in China

**DOI:** 10.3389/fpubh.2024.1469980

**Published:** 2024-12-11

**Authors:** Xianghong Ding, Zijuan Shi, Liping Xiang, Qin Liu, Li Wu, Qingwen Long, Yujun Lee

**Affiliations:** ^1^Mental Health Center, Affiliated Hospital of North Sichuan Medical College, Nanchong, China; ^2^School of Nursing, North Sichuan Medical College, Nanchong, China; ^3^Department of Neurology, Affiliated Hospital of North Sichuan Medical College, Nanchong, China; ^4^Department of Foreign Languages and Culture, North Sichuan Medical College, Nanchong, China

**Keywords:** heart disease, depression, nomogram, predictive model, risk factors

## Abstract

**Background:**

Comorbid depression, frequently observed in heart disease patients, has detrimental effects on mental health and may exacerbate cardiac conditions. The objective of this study was to create and validate a risk prediction nomogram specifically for comorbid depression in older adult patients suffering from heart disease.

**Methods:**

The 2018 data from the Chinese Longitudinal Healthy Longevity Survey (CLHLS) was analyzed and 2,110 older adult patients with heart disease aged 60 and above were included in the study. They were randomly divided in a 7:3 ratio into a training set (*n* = 1,477) and a validation set (*n* = 633). Depression symptoms were assessed using the 10-item Center for Epidemiologic Studies Depression Scale (CESD-10) and the participants were categorized into depressed (*n* = 687) and non-depressed (*n* = 1,423) groups. We collected information regarding general demographics, lifestyle habits, and medical history of the included patients. LASSO regression and binary logistic regression analyses were performed to identify independent risk factors and construct the depression prediction nomogram. Receiver operating characteristic curve analysis and the Hosmer-Lemeshow test were used to assess the model's discrimination and calibration. Decision curve analysis helped evaluate the clinical utility of the predictive nomogram.

**Results:**

Based on the LASSO and multivariable regression analyses, education level, quality of life, sleep quality, frequency of watching TV, and history of arthritis were identified as independent risk factors for comorbid depression in the older adult heart disease patients. A nomogram model was constructed with these five independent risk factors. The nomogram showed good clinical performance with an area under the curve (AUC) value of 0.816 (95% CI: 0.793 to 0.839). The calibration curves and Hosmer-Lemeshow goodness-of-fit test (training set $\chi_{t}^{2}$ = 4.796, *p* = 0.760; validation set $\chi_{v}^{2}$ = 7.236, *p* = 0.511) showed its satisfactory. Clinical usefulness of the nomogram was confirmed by decision curve analysis.

**Conclusions:**

A five-parameter nomogram for predicting depression in older adult heart disease patients was developed and validated. The nomogram demonstrated high accuracy, discrimination ability, and clinical utility in assessing the risk of depression in the older adult patients with heart disease.

## 1 Introduction

Heart diseases are highly prevalent among the older adults and are a significant cause of mortality in this demographic (1). Geriatric cardiology encompasses clinical diagnosis of structural heart abnormalities such as congenital heart defects, valvular heart diseases, and cardiomyopathies, as well as non-structural heart issues such as arrhythmias. These conditions are manifested with symptoms such as dyspnea, fatigue, and asthenia, and significantly impact the daily activities and functional capacity of the older adults (2, 3). Depression is a common mental disorder among older adult people, characterized by symptoms such as persistent sadness, anhedonia, sleep disturbances, and suicidal ideation (4). The physiological and psychological faculties of the older adults gradually deteriorate with age because of reduced nervous system metabolism, alterations in the neurotransmitters, and changes in the health status and family life. This leads to melancholy, a loss of zest for life, and shifts in self-perception and physical capabilities. Several studies have reported that older adult individuals with heart disease are at a higher risk of depression compared to the general older adult population (5). with a complex reciprocal influence observed between the two conditions (6, 7). The co-occurrence of depression and heart disease can lead to a mutually exacerbating cycle through various mechanisms. Depression is associated with increased severity of heart disease and the likelihood of rehospitalization and mortality, and reduced treatment adherence and the quality of life of patients. The interplay between heart disease and depression is complex (8), and involves neurobiological mechanisms, including a complex interplay between the neuroendocrine system, inflammatory processes, and psychosocial factors. Chronic stress and anxiety are common in patients with heart disease and activates the hypothalamic-pituitary-adrenal (HPA) axis. This increases cortisol levels and impacts cardiac function and vascular integrity (9). Furthermore, depressive mood disrupts the balance of the autonomic nervous system, reduces the heart rate variability (10), and increases the risk of cardiac incidents. Patients with heart disease (11–14) are significantly more likely to suffer from depression than those without heart disease. Among older adult individuals, those with both heart disease and depression face a higher all-cause mortality rate than those with only one of these conditions (15). The risk of myocardial infarction is twice as high in patients with depression compared to the general population, while the incidence of depression among myocardial infarction patients is three times higher than that in the general population (16, 17). Cardiovascular disease and comorbid depression are pressing public health issues. The “2022 Report on Cardiovascular Health and Diseases in China” indicates an increasing prevalence of cardiovascular diseases in China (18). Over 80% of coronary heart disease patients experience anxiety and depression (19). Depression, a leading psychological disorder among the older adults, significantly impacts quality of life and places a considerable burden on healthcare systems (20).

Previous studies (21, 22) have focused on the determinants of depression in the older adults with cognitive disorders or at an advanced age in China, but only few studies have investigated older adult patients with heart disease. The present study used the national data from the eighth wave of the Chinese Longitudinal Healthy Longevity Survey (CLHLS) and analyzed various indicators, including demographic data, lifestyle habits, and medical history of older adult individuals aged ≥ 60 years with heart disease. The main objective of this study was to identify the risk factors for depression and to develop and validate a predictive nomogram model for the older adult heart disease patients.

## 2 Materials and methods

### 2.1 Data sources

**Figure 1:**
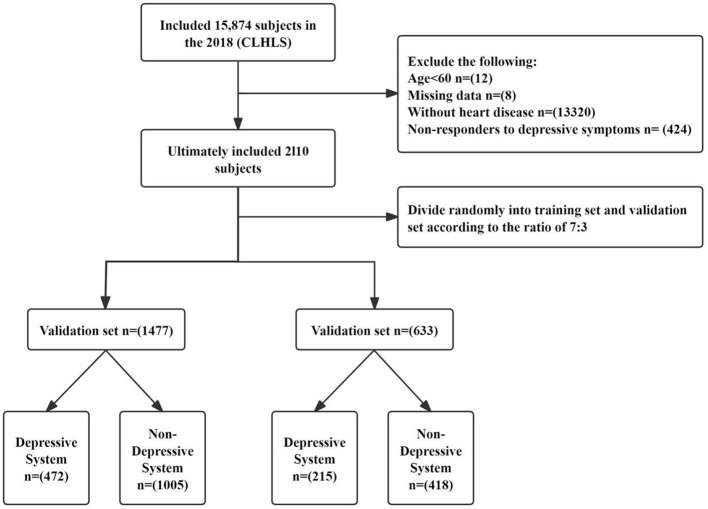
Flow diagram of the study design.

This study utilized data from the 2018 cross-sectional Chinese Longitudinal Healthy Longevity Survey (CLHLS), which provides comprehensive information on the health and quality of life of older adult individuals across 23 provinces in China. The survey, conducted from 1998 to 2018, amassed a sample size of 113,000 participants, making it the country's largest social science survey. Informed consent was obtained from all participants, and ethical approval was granted by the relevant review boards. Survey personnel were registered and authorized to access the data platform. The primary subjects of the 2018 CLHLS data were individuals aged ≥65 years, although a small number of individuals younger than 65 years were also included. Following World Health Organization guidelines, individuals aged ≥60 years are classified as older adults in developing countries, while those aged ≥65 years are considered older adults in developed countries (23). As China is classified as a developing country, we defined the older adult population as individuals aged ≥60 years. The inclusion criteria for this study were as follows: (1) age ≥60 years; (2) self-reported, physician-diagnosed heart disease with a clear confirmation of diagnosis; (3) completion of all 10 items on the Center for Epidemiological Studies Depression Scale (CES-D) with valid responses; (4) no missing data for key variables. The initial sample consisted of 15,874 older adult individuals, but after excluding individuals aged under 60 years (*n* = 12), those with missing data (*n* = 8), those without heart disease (*n* = 13,320), and those not responding to depression symptoms (*n* = 424), the final sample for model construction included 2,110 older adult patients with heart disease (Figure 1).

### 2.2 Variables included in the study

Depressive symptoms of the study participants in 2018 were evaluated using the 10-item Center for Epidemiological Studies Depression Scale (CESD-10) (24). This scale is comprised of 10 items, including eight items on negative experiences and 2 items on positive experiences during the past week. The patients were inquired about the frequency of nine affective states and sleep quality over the past week. The responses for each item were rated on a 4-point Likert scale and the scores for each item ranged from 0 to 3 (with reverse scoring for negative states). The total score ranged from 0 to 30 with higher scores reflecting increased severity of the depressive symptoms (25). This scale is used for preliminary screening of subthreshold depression epidemiology and has good reliability and validity in the older adult population (26). Based on previous studies (27–29), we used a cutoff score of 10 (≥10) to identify patients with depression.

Data were collected by trained community physicians or public health workers through face-to-face interviews at respondents' homes. All disease histories were confirmed by hospital diagnoses. Smoking was defined as current smoking, encompassing both daily and occasional smokers. Drinking referred to individuals with an ongoing drinking habit. Household income was categorized as follows: less than 10,000 RMB was classified as very poor, 10,000–29,999 RMB as poor, 30,000–49,999 RMB as moderate, and over 50,000 RMB as wealthy (30, 31). The education level was categorized into three groups: illiterate (never attended school), primary school (1–6 years), and middle school or higher (≥7 years). Marital status was classified into four categories: married, divorced, widowed, and other (including those married but not living with their spouse, as well as those never married). Sleep quality, based on the question “How many hours of sleep do you usually get each day?”, was divided into five levels: very poor (<4 h), poor (4–5.9 h), average (6–7.9 h), good (8–9.9 h), and very good (>10 h). Responses to the question “How do you feel about your current life?” were classified as good, average, or not good. Living arrangements were classified into three categories: living with family, living alone, and residing in an institutional setting. Other variables, such as pension status, financial support from children, and annual physical examinations, were self-reported by participants with binary responses (yes/no), similar to various medical histories. After a comprehensive literature review, additional variables were included in the study based on their potential influence on comorbid depression in older adult patients with heart disease (Table 1). All variables had a missing data rate of less than 15%, with the highest missing value percentage being for education (11.85%). To improve the accuracy of model analysis, multiple imputation techniques were applied to address missing data.

**Table 1:** Categorical variable assignment.

**Variable**	**Variable assignment**
Sex	Male = 1; female = 2
Age	(60-69) = 1; (70–79) = 2; (80-89) = 3; (≥90) = 4
Education level	Illiterate = 1; primary school = 2; middle school or above = 3
Marital status	Married = 1; divorced = 2; widowed = 3; others = 4
Current place of residence	City = 1; town = 2; rural = 3
Annual family income	Very poor = 1; poor = 2; middle = 3; rich = 4
Living arrangement	Family = 1; alone = 2; institution = 3
Medical insurance status	Yes = 1; no = 0
Pension status	Yes = 1; no = 0
Number of children	(0) = 1; (1–3) = 2; (>3) = 3
Financial support from children	Yes = 1; no = 0
Community services	Yes = 1; no = 0
Quality of life	Good = 1; moderate = 2; poor = 3
Fall	Yes = 1; no = 0
Sleep quality	Excellent = 1; good = 2; moderate = 3; poor = 4; very poor = 5
Current smoking status	Yes = 1; no = 0
Current alcohol status	Yes = 1; no = 0
Annual physical examination	Yes = 1; no = 0
TV (watching frequency)	Daily = 1; weekly = 2; monthly = 3; occasionally = 4; never = 5
Different medical histories	Yes = 1; no = 0

### 2.3 Statistical analysis

Categorical variables were expressed as counts and percentages. Inter-group differences were assessed using the chi-square test or Fisher's exact test, as appropriate. The least absolute shrinkage and selection operator (LASSO) regression was used to identify risk factors associated with depression in older adult patients with heart disease. LASSO regression applies an L1 penalty term that shrinks the coefficients of less relevant variables toward zero, facilitating effective variable selection. To enhance the clarity of the results, model predictions were visualized using nomograms, which provide an intuitive representation of how variables influence risk, aiding in both risk assessment and decision-making. The predictive performance of the model was evaluated using receiver operating characteristic (ROC) curves, with accuracy measured by the area under the curve (AUC). Model calibration was assessed using the Hosmer-Lemeshow test, and internal validation was performed with 1,000 bootstrap resampling iterations to generate a calibration plot. Decision curve analysis (DCA) was employed to assess the clinical utility of the predictive nomogram. All statistical analyses were performed using R software (version 4.3.1), with a two-tailed *P* < 0.05 considered statistically significant.

## 3 Results

### 3.1 Comparison of basic and clinical characteristics

Among the 2,110 older adult patients with heart disease included in this study, 861 were male and 1,249 were female. The prevalence rate of depression was 32.56%. The participants were randomly assigned in a 7:3 ratio into a training group (*n* = 1,477) and a validation group (*n* = 633). The prevalence rates of depression in the training and validation groups were 31.96% and 33.97%, respectively. As shown in Tables 2, 3, there were no statistically significant differences in the basic and clinical characteristics between the two groups (*p* > 0.05) except for the health insurance status and history of rheumatic diseases. In the training group, we observed statistically significant differences in the prevalence rates of depression across various demographics, including gender, education level, household income, marital status, cohabitation arrangements, number of children, quality of life, history of falls, sleep quality, frequency of annual health examinations, television watching habits, and medical history of diabetes, respiratory diseases, gastrointestinal ulcers, parkinson's disease, arthritis, and chronic nephritis (*p* < 0.05). In the validation group, we observed significant differences in the prevalence rates of depression across various characteristics such as gender, age, education level, household income, cohabitation arrangements, quality of life, history of falls, sleep quality, television watching habits, and medical history of diabetes, stroke, respiratory diseases, arthritis, and rheumatic diseases (*p* < 0.05).

**Table 2:** General information and lifestyle habits questionnaire for participants in the training and validation data sets.

**Variable**	**Training group**, ***n*** **(%)**				**Validation group**, ***n*** **(%)**				***P***
	**Total (*****n*** = **1,477)**	**No DS (*****n*** = **1005)**	**DS (*****n*** = **472)**	***P***	**Total (*****n*** = **633)**	**No DS (*****n*** =**418)**	**DS (*****n*** = **215)**	***P***	
**Sex**				<0.001^**^				0.029^*^	0.713
Male	607 (41.10)	446 (44.38)	161 (34.11)		254 (40.13)	181 (43.30)	73 (33.95)		
Female	870 (58.90)	559 (55.62)	311 (65.89)		379 (59.87)	237 (56.70)	142 (66.05)		
**Age, years**				0.224				0.021^*^	0.942
60~	139 (9.41)	104 (10.35)	35 (7.42)		64 (10.11)	50 (11.96)	14 (6.51)		
70~	462 (31.28)	319 (31.74)	143 (30.30)		197 (31.12)	130 (31.10)	67 (31.16)		
80~	474 (32.09)	312 (31.04)	162 (34.32)		197 (31.12)	116 (27.75)	81 (37.67)		
90~	402 (27.22)	270 (26.87)	132 (27.97)		175 (27.65)	122 (29.19)	53 (24.65)		
**Education level**				<0.001^**^				<0.001^**^	0.441
Illiterate	568 (38.46)	338 (33.63)	230 (48.73)		225 (35.55)	127 (30.38)	98 (45.58)		
Primary school	465 (31.48)	340 (33.83)	125 (26.48)		211 (33.33)	156 (37.32)	55 (25.58)		
Middle school or above	444 (30.06)	327 (32.54)	117 (24.79)		197 (31.12)	135 (32.30)	62 (28.84)		
**Current place of residence**				0.988				0.943	0.11
City	630 (42.65)	430 (42.79)	200 (42.37)		241 (38.07)	160 (38.28)	81 (37.67)		
Town	376 (25.46)	255 (25.37)	121 (25.64)		183 (28.91)	119 (28.47)	64 (29.77)		
Rural	471 (31.89)	320 (31.84)	151 (31.99)		209 (33.02)	139 (33.25)	70 (32.56)		
**Annual family income**				0.049^*^				0.029^*^	0.619
Very poor	384 (26.00)	240 (23.88)	144 (30.51)		165 (26.07)	94 (22.49)	71 (33.02)		
Poor	215 (14.56)	154 (15.32)	61 (12.92)		106 (16.75)	70 (16.75)	36 (16.74)		
Middle	218 (14.76)	149 (14.83)	69 (14.62)		90 (14.22)	65 (15.55)	25 (11.63)		
Rich	660 (44.69)	462 (45.97)	198 (41.95)		272 (42.97)	189 (45.22)	83 (38.60)		
**Marital status**				0.002^**^				0.183	0.318
Married	669 (45.29)	486 (48.36)	183 (38.77)		285 (45.02)	199 (47.61)	86 (40.00)		
Divorced	8 (0.54)	7 (0.70)	1 (0.21)		334 (52.76)	210 (50.24)	124 (57.67)		
Widowed	766 (51.86)	489 (48.66)	277 (58.69)		14 (2.21)	9 (2.15)	5 (2.33)		
Others	34 (2.30)	23 (2.29)	11 (2.33)						
**Living arrangement**				0.009^**^				0.022^*^	0.955
Family	1150 (77.86)	804 (80.00)	346 (73.31)		490 (77.41)	337 (80.62)	153 (71.16)		
Alone	243 (16.45)	153 (15.22)	90 (19.07)		105 (16.59)	61 (14.59)	44 (20.47)		
Institution	84 (5.69)	48 (4.78)	36 (7.63)		38 (6.00)	20 (4.78)	18 (8.37)		
**Medical insurance status**				0.461				0.68	0.039^*^
Yes	1267 (85.78)	857 (85.27)	410 (86.86)		520 (82.15)	341 (81.58)	179 (83.26)		
No	210 (14.22)	148 (14.73)	62 (13.14)		113 (17.85)	77 (18.42)	36 (16.74)		
**Pension status**				0.501				0.928	0.287
Yes	643 (43.53)	444 (44.18)	199 (42.16)		259 (40.92)	170 (40.67)	89 (41.40)		
No	834 (56.47)	561 (55.82)	273 (57.84)		374 (59.08)	248 (59.33)	126 (58.60)		
**Number of children**				<0.001^**^				0.071	0.84
0	26 (1.76)	14 (1.39)	12 (2.54)		9 (1.42)	4 (0.96)	5 (2.33)		
1-3	730 (49.42)	532 (52.94)	198 (41.95)		311 (49.13)	217 (51.91)	94 (43.72)		
>3	721 (48.82)	459 (45.67)	262 (55.51)		313 (49.45)	197 (47.13)	116 (53.95)		
**Financial support from children**				0.102				0.193	0.929
Yes	518 (35.07)	338 (33.63)	180 (38.14)		224 (35.39)	140 (33.49)	84 (39.07)		
No	959 (64.93)	667 (66.37)	292 (61.86)		409 (64.61)	278 (66.51)	131 (60.93)		
**Community services**				0.647				0.223	>0.999
Yes	994 (67.30)	672 (66.87)	322 (68.22)		426 (67.30)	274 (65.55)	152 (70.70)		
No	483 (32.70)	333 (33.13)	150 (31.78)		207 (32.70)	144 (34.45)	63 (29.30)		
**Quality of life**				<0.001^**^				<0.001^**^	0.938
Good	1015 (68.72)	805 (80.10)	210 (44.49)		439 (69.35)	330 (78.95)	109 (50.70)		
Moderate	402 (27.22)	183 (18.21)	219 (46.40)		170 (26.86)	85 (20.33)	85 (39.53)		
Poor	60 (4.06)	17 (1.69)	43 (9.11)		24 (3.79)	3 (0.72)	21 (9.77)		
**Fall**				0.01^*^				0.002^**^	0.698
Yes	353 (23.90)	220 (21.89)	133 (28.18)		157 (24.80)	87 (20.81)	70 (32.56)		
No	1,124 (76.10)	785 (78.11)	339 (71.82)		476 (75.20)	331 (79.19)	145 (67.44)		
**Sleep quality**				<0.001^**^				<0.001^**^	0.51
Excellent	221 (14.96)	200 (19.90)	21 (4.45)		87 (13.74)	77 (18.42)	10 (4.65)		
Good	479 (32.43)	400 (39.80)	79 (16.74)		192 (30.33)	165 (39.47)	27 (12.56)		
Moderate	470 (31.82)	287 (28.56)	183 (38.77)		202 (31.91)	117 (27.99)	85 (39.53)		
Poor	262 (17.74)	98 (9.75)	164 (34.75)		128 (20.22)	51 (12.20)	77 (35.81)		
Very poor	45 (3.05)	20 (1.99)	25 (5.30)		24 (3.79)	8 (1.91)	16 (7.44)		
**Current smoking status**				0.47				0.605	0.953
Yes	171 (11.58)	121 (12.04)	50 (10.59)		72 (11.37)	50 (11.96)	22 (10.23)		
No	1,306 (88.42)	884 (87.96)	422 (89.41)		561 (88.63)	368 (88.04)	193 (89.77)		
**Current alcohol status**				0.429				0.138	0.573
Yes	156 (10.56)	111 (11.04)	45 (9.53)		61 (9.64)	46 (11.00)	15 (6.98)		
No	1,321 (89.44)	894 (88.96)	427 (90.47)		572 (90.36)	372 (89.00)	200 (93.02)		
**Annual physical examination**				0.033^*^				0.457	>0.999
Yes	1,111 (75.22)	773 (76.92)	338 (71.61)		476 (75.20)	310 (74.16)	166 (77.21)		
No	366 (24.78)	232 (23.08)	134 (28.39)		157 (24.80)	108 (25.84)	49 (22.79)		
**TV (watching frequency)**				<0.001^**^				0.001^**^	0.518
Daily	997 (67.50)	738 (73.43)	259 (54.87)		412 (65.09)	296 (70.81)	116 (53.95)		
Weekly	122 (8.26)	67 (6.67)	55 (11.65)		55 (8.69)	30 (7.18)	25 (11.63)		
Monthly	39 (2.64)	20 (1.99)	19 (4.03)		23 (3.63)	13 (3.11)	10 (4.65)		
Occasionally	40 (2.71)	25 (2.49)	15 (3.18)		23 (3.63)	14 (3.35)	9 (4.19)		
Never	279 (18.89)	155 (15.42)	124 (26.27)		120 (18.96)	65 (15.55)	55 (25.58)		

DS, depressive symptom; *P, p*-value; ^*^p < 0.05; ^**^p < 0.01.

**Table 3:** Diseases history questionnaire for participants in the training and validation datasets.

**Variable**	**Training group**, ***n*** **(%)**				**Validation group**, ***n*** **(%)**				***P***
	**Total (*****n*** = **1,477)**	**No DS (*****n*** = **1,005)**	**DS (*****n*** = **472)**	***P***	**Total (*****n*** = **633)**	**No DS (*****n*** = **418)**	**DS (*****n*** = **215)**	***P***	
**Hypertension**				0.915				0.789	0.36
Yes	950 (64.32)	645 (64.18)	305 (64.62)		421 (66.51)	276 (66.03)	145 (67.44)		
No	527 (35.68)	360 (35.82)	167 (35.38)		212 (33.49)	142 (33.97)	70 (32.56)		
**Diabetes mellitus**				0.031^*^				0.002^**^	0.935
Yes	312 (21.12)	196 (19.50)	116 (24.58)		132 (20.85)	72 (17.22)	60 (27.91)		
No	1165 (78.88)	809 (80.50)	356 (75.42)		501 (79.15)	346 (82.78)	155 (72.09)		
**History of stroke**				0.054				<0.001^**^	0.15
Yes	329 (22.27)	209 (20.80)	120 (25.42)		160 (25.28)	80 (19.14)	80 (37.21)		
No	1148 (77.73)	796 (79.20)	352 (74.58)		473 (74.72)	338 (80.86)	135 (62.79)		
**Respiratory disease**				<0.001^**^				0.006^**^	0.359
Yes	281 (19.03)	165 (16.42)	116 (24.58)		109 (17.22)	59 (14.11)	50 (23.26)		
No	1196 (80.97)	840 (83.58)	356 (75.42)		524 (82.78)	359 (85.89)	165 (76.74)		
**Tuberculosis**				0.925				0.381	>0.999
Yes	29 (1.96)	19 (1.89)	10 (2.12)		13 (2.05)	7 (1.67)	6 (2.79)		
No	1448 (98.04)	986 (98.11)	462 (97.88)		620 (97.95)	411 (98.33)	209 (97.21)		
**Cataract**				0.4				0.31	0.947
Yes	363 (24.58)	240 (23.88)	123 (26.06)		154 (24.33)	96 (22.97)	58 (26.98)		
No	1114 (75.42)	765 (76.12)	349 (73.94)		479 (75.67)	322 (77.03)	157 (73.02)		
**Glaucoma**				0.766				>0.999	0.98
Yes	58 (3.93)	41 (4.08)	17 (3.60)		24 (3.79)	16 (3.83)	8 (3.72)		
No	1419 (96.07)	964 (95.92)	455 (96.40)		609 (96.21)	402 (96.17)	207 (96.28)		
**Cancer**				>0.999				0.402	>0.999
Yes	31 (2.10)	21 (2.09)	10 (2.12)		14 (2.21)	11 (2.63)	3 (1.40)		
No	1446 (97.90)	984 (97.91)	462 (97.88)		619 (97.79)	407 (97.37)	212 (98.60)		
**Gastrointestinal ulcer**				0.001^**^				0.062	0.509
Yes	123 (8.33)	67 (6.67)	56 (11.86)		59 (9.32)	32 (7.66)	27 (12.56)		
No	1354 (91.67)	938 (93.33)	416 (88.14)		574 (90.68)	386 (92.34)	188 (87.44)		
**Parkinson**				0.047^*^				0.177	0.303
Yes	14 (0.95)	13 (1.29)	1 (0.21)		10 (1.58)	9 (2.15)	1 (0.47)		
No	1463 (99.05)	992 (98.71)	471 (99.79)		623 (98.42)	409 (97.85)	214 (99.53)		
**Bedsore**				>0.999				0.172	0.861
Yes	9 (0.61)	6 (0.60)	3 (0.64)		5 (0.79)	5 (1.20)	0 (0.00)		
No	1468 (99.39)	999 (99.40)	469 (99.36)		628 (99.21)	413 (98.80)	215 (100.00)		
**Arthritis**				<0.001^**^				0.001^**^	0.222
Yes	286 (19.36)	158 (15.72)	128 (27.12)		138 (21.80)	74 (17.70)	64 (29.77)		
No	1191 (80.64)	847 (84.28)	344 (72.88)		495 (78.20)	344 (82.30)	151 (70.23)		
**Dementia**				0.577				0.097	0.554
Yes	17 (1.15)	10 (1.00)	7 (1.48)		10 (1.58)	4 (0.96)	6 (2.79)		
No	1460 (98.85)	995 (99.00)	465 (98.52)		623 (98.42)	414 (99.04)	209 (97.21)		
**Epilepsy**				0.185				>0.999	0.23
Yes	6 (0.41)	6 (0.60)	0 (0.00)		6 (0.95)	4 (0.96)	2 (0.93)		
No	1471 (99.59)	999 (99.40)	472 (100.00)		627 (99.05)	414 (99.04)	213 (99.07)		
**Biliary tract disease**				0.658				0.556	0.262
Yes	123 (8.33)	81 (8.06)	42 (8.90)		63 (9.95)	39 (9.33)	24 (11.16)		
No	1354 (91.67)	924 (91.94)	430 (91.10)		570 (90.05)	379 (90.67)	191 (88.84)		
**Dyslipidemia**				0.333				0.39	>0.999
Yes	214 (14.49)	139 (13.83)	75 (15.89)		91 (14.38)	56 (13.40)	35 (16.28)		
No	1263 (85.51)	866 (86.17)	397 (84.11)		542 (85.62)	362 (86.60)	180 (83.72)		
**Rheumatism**				0.18				0.015^*^	0.019^*^
Yes	113 (7.65)	70 (6.97)	43 (9.11)		69 (10.90)	36 (8.61)	33 (15.35)		
No	1364 (92.35)	935 (93.03)	429 (90.89)		564 (89.10)	382 (91.39)	182 (84.65)		
**Chronic nephritis**				0.03^*^				0.982	0.886
Yes	41 (2.78)	21 (2.09)	20 (4.24)		19 (3.00)	12 (2.87)	7 (3.26)		
No	1436 (97.22)	984 (97.91)	452 (95.76)		614 (97.00)	406 (97.13)	208 (96.74)		
**Hepatitis**				>0.999				0.172	0.429
Yes	6 (0.41)	4 (0.40)	2 (0.42)		5 (0.79)	5 (1.20)	0 (0.00)		
No	1471 (99.59)	1001 (99.60)	470 (99.58)		628 (99.21)	413 (98.80)	215 (100.00)		

DS, depressive symptom; P, p-value; ^*^p < 0.05; ^**^p < 0.01.

### 3.2 Variable selection and prediction model construction using LASSO regression analyses

**Figure 2:**
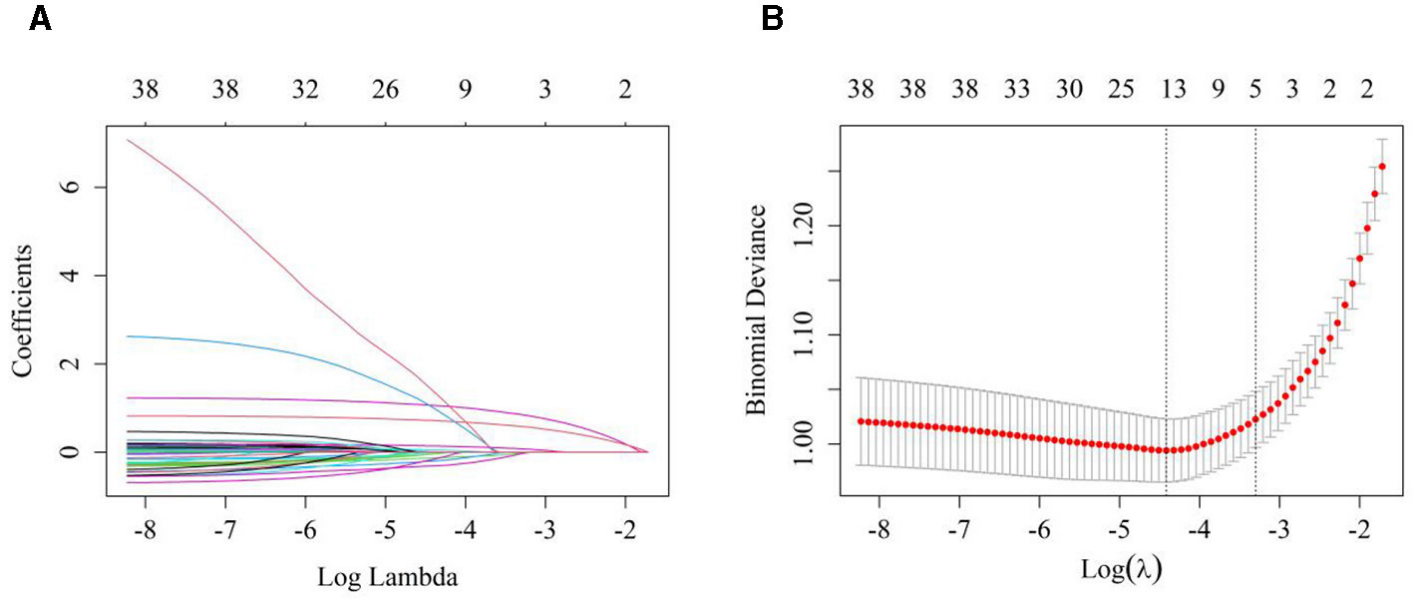
Variable selection using the LASSO regression model. **(A)** LASSO coefficient pathway diagram of predictive variables, each distinct colored line represents the trajectory of a feature's coefficient, as it evolves with the increase in the regularization parameter λ. As λ increases, certain coefficients gradually diminish and, ultimately, approach zero, signifying that these features are effectively pruned from the model by the LASSO regression. **(B)** A tenfold cross-validation was performed to select the optimal penalty parameter (lambda) for the model. The cross-validation identified log(lambda.1se) = −3.392 (lambda.1se = 0.03364981) as the point where the model error was within one standard deviation of the minimum.

The LASSO regression model was used for dimensionality reduction based on data from the training group. Tenfold cross-validation was employed to determine the optimal penalty parameter (lambda). Cross-validation identified log(lambda.1se) = −3.392 (lambda.1se = 0.03364981) as the point where the model error was within one standard deviation of its minimum value. The variables with non-zero coefficients included education level, life satisfaction, sleep quality, frequency of TV watching, and history of arthritis (Figure 2). Multifactorial logistic regression analyses revealed that education level, life satisfaction, sleep quality, frequency of TV watching, and history of arthritis were independent risk factors for comorbid depression in older adult patients with heart disease (*p* < 0.05; Table 4).

**Table 4:** Logistic regression analysis of depression in older adult patients with heart disease.

**Variable**	**β Value**	***S_*t*_***	***Z***	***OR(95%CI)***	***P***
Intercept	−2.185	0.303	−7.214	0.113 (0.062–0.204)	<0.001^**^
**Education (reference** = **Illiterate)**					
Primary school	−0.382	0.163	−2.337	0.682 (0.495–0.94)	0.019^*^
Middle school or above	−0.437	0.17	−2.566	0.646 (0.462–0.902)	0.01^*^
**Life quality (reference** = **good)**					
Moderate	1.417	0.141	10.029	4.123 (3.126–5.437)	<0.001^**^
Poor	1.592	0.333	4.778	4.915 (2.558–9.443)	<0.001^**^
**Sleep quality (reference** = **very good)**					
Fairly good	0.465	0.272	1.71	1.591 (0.934–2.711)	0.087
Average	1.652	0.26	6.364	5.219 (3.138–8.682)	<0.001^**^
Poor	2.462	0.278	8.865	11.725 (6.803–20.206)	<0.001^**^
Very poor	2.128	0.413	5.156	8.394 (3.739–18.844)	<0.001^**^
**TV (reference** = **daily)**					
Weekly	0.881	0.235	3.744	2.413 (1.522–3.827)	<0.001^**^
Monthly	1.006	0.384	2.621	2.734 (1.289–5.802)	0.009^**^
Occasionally	0.568	0.38	1.495	1.764 (0.838–3.712)	0.135
Never	0.737	0.178	4.144	2.089 (1.474–2.96)	<0.001^**^
**History of arthritis (reference** = **yes)**					
No	−0.559	0.16	−3.492	0.572 (0.418–0.782)	<0.001^**^

St, standard error; Z, z-score; OR, odds ratio; CI, confidence interval; P, p-value; ^*^p < 0.05; ^**^p < 0.01.

### 3.3 Construction of the nomogram predictive model

**Figure 3:**
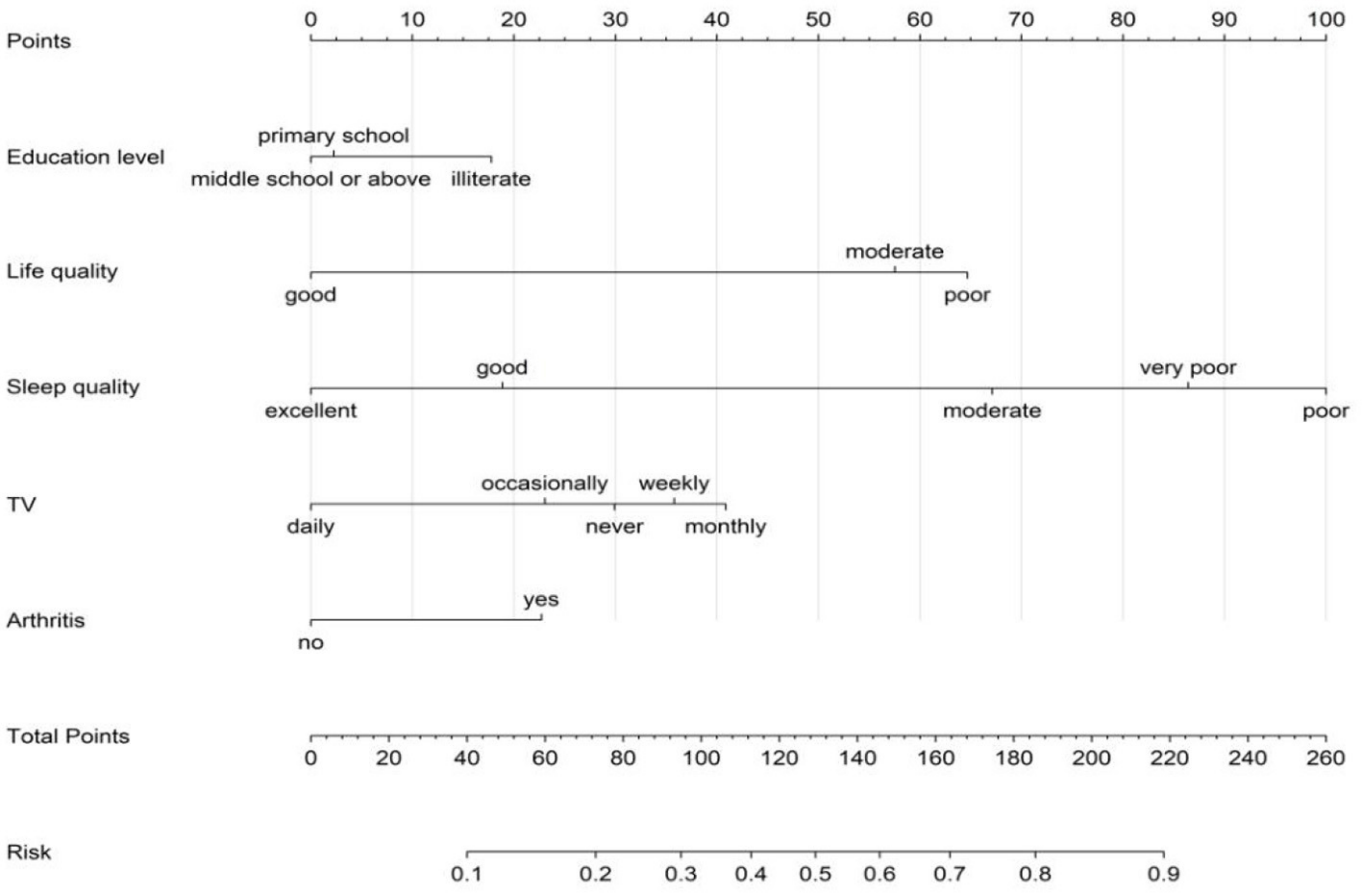
Nomogram for predicting the risk of depression in older adult patients with heart disease.

A nomogram predictive model for depression in older adult patients with heart disease was constructed for the training group with the five independent risk factors, namely, education level, life quality, sleep quality, frequency of TV watching, and history of arthritis (Figure 3). In this model, each significant variable was assigned a weighted score ranging from 0 to 100. The total score was estimated by adding the scores for each risk factor in the nomogram. The probability of depression was predicted in the older adult patients with heart disease based on the total score.

### 3.4 Validation of the nomogram predictive model

**Figure 4:**
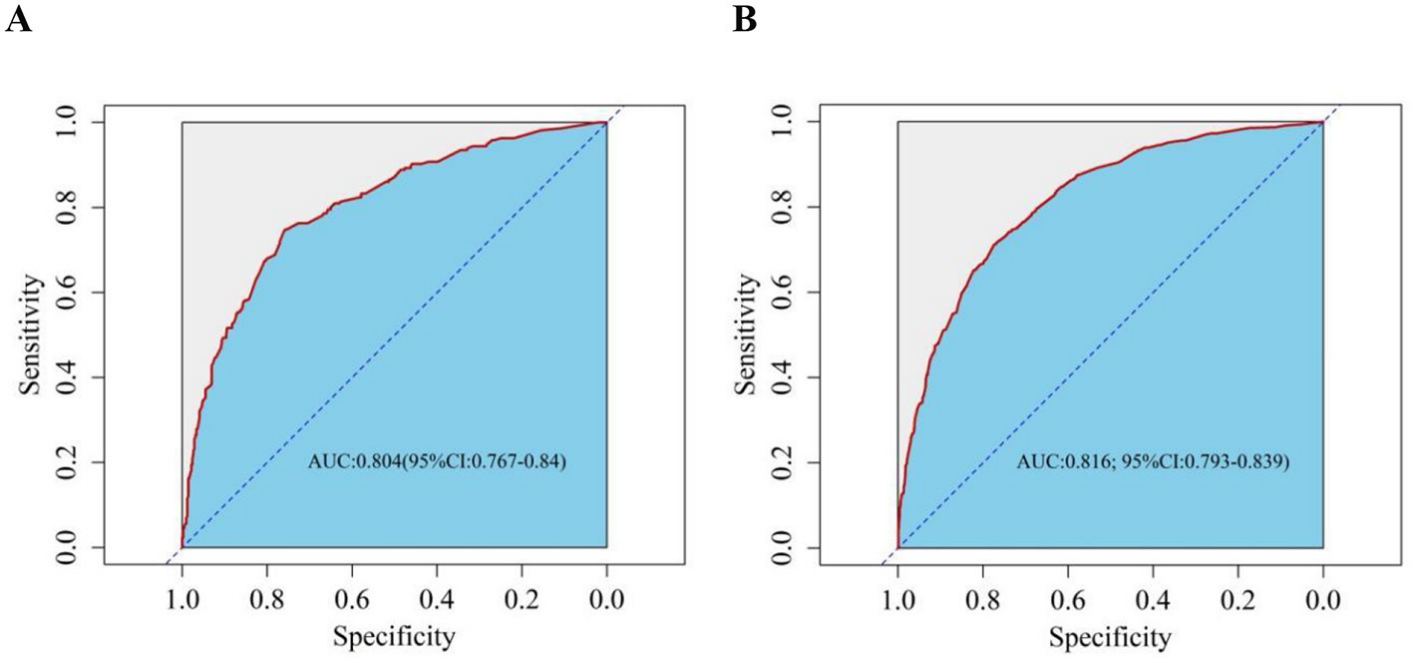
Receiver operating characteristic (ROC) curve evaluates the risk prediction nomogram for depression in older adult patients with heart disease. The Y-axis represents the true-positive rate of the risk prediction, and the X-axis represents the false-positive rate of risk prediction. **(A)** ROC curve of the training set; **(B)** ROC curve of the validation set.

**Figure 5:**
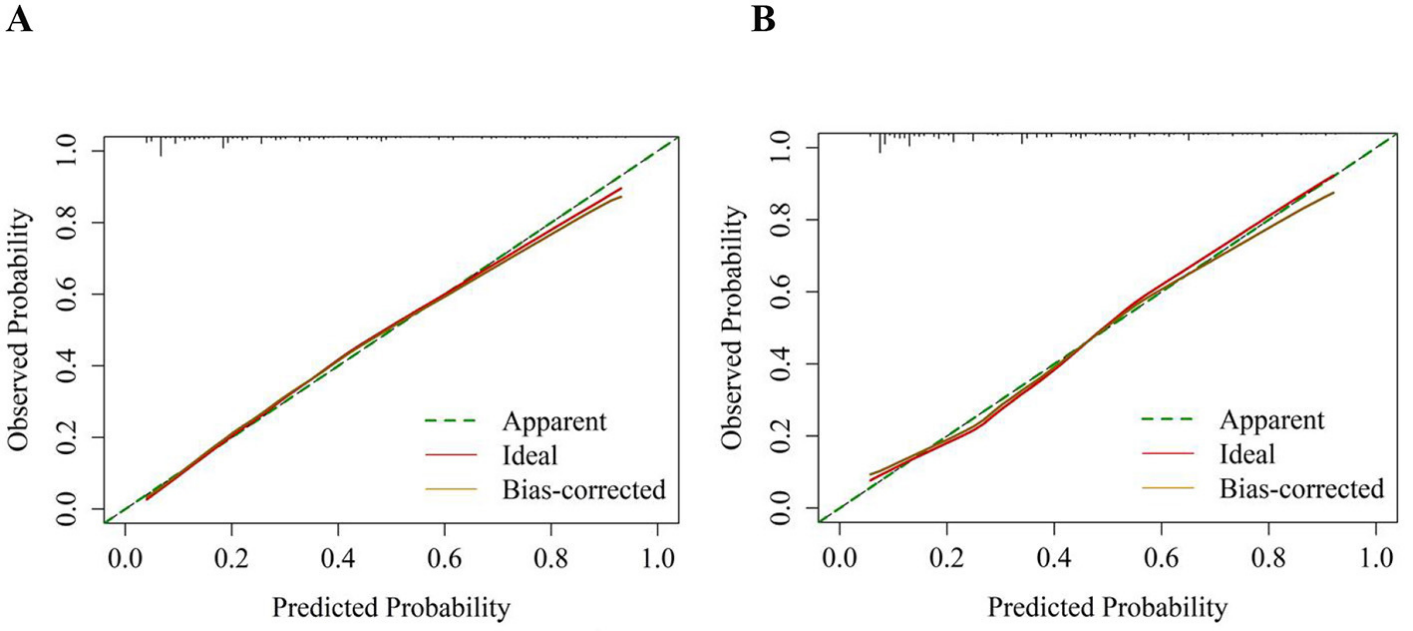
Calibration curves for the risk nomogram of depression in older adult patients with heart disease. The Y-axis represents the actual number of diagnosed depression cases, and the X-axis represents the predictive risk of depression. The diagonal dotted line represents perfect prediction by an ideal model, and a closer fit to the diagonal dotted line indicates better prediction. **(A)** Calibration curve of the training set; **(B)** Calibration curve of the validation set.

**Figure 6:**
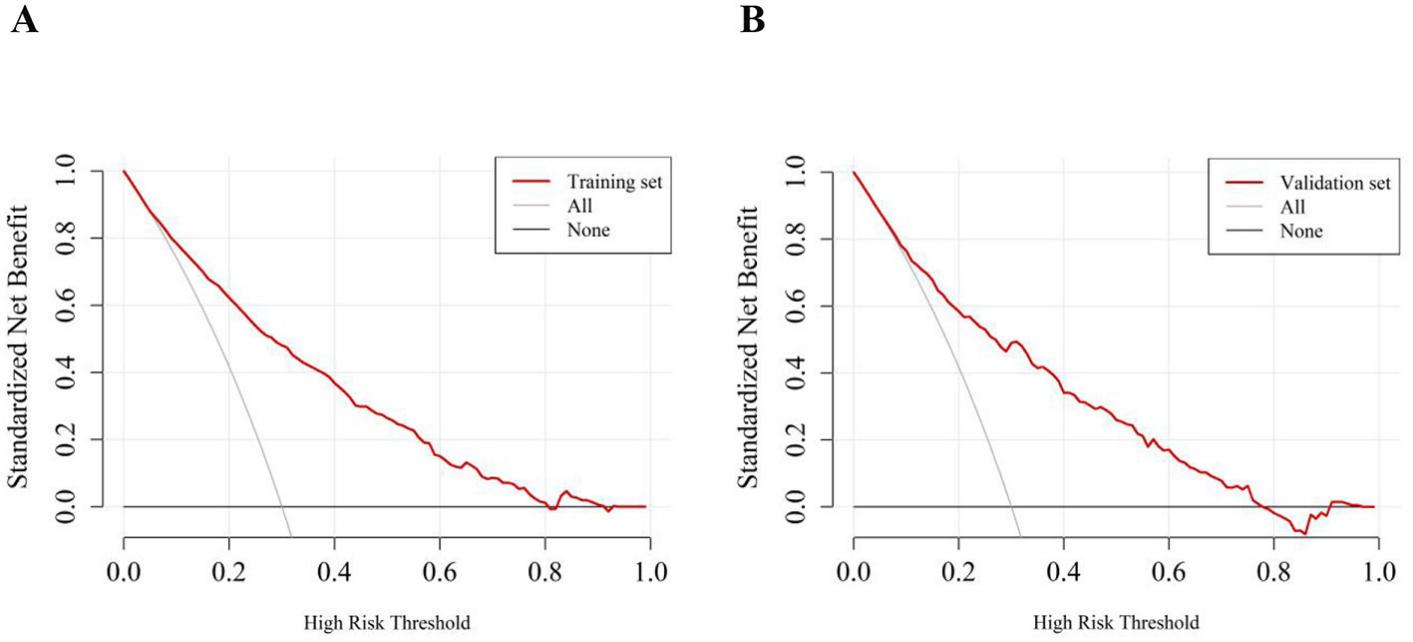
Decision curve analysis for the risk nomogram of depression in older adult patients with heart disease. The Y-axis represents the net benefit. The thick solid line represents the assumption that none of the patients have depression, the thin solid line represents the assumption that all patients have depression, and the red line represents the risk nomogram. **(A)** Decision curve of the training set; **(B)** Decision curve of the validation set.

Next, we performed internal validation of the nomogram predictive model for depression in the older adult patients with heart disease. ROC curve analysis results showed good predictive power for the nomogram with AUC values of 0.816 (95% *CI:* 0.793–0.839) and 0.804 (95% *CI:* 0.767–0.840) for the training and validation groups, respectively. The predictive model was then validated using the bootstrap method with 1,000 resampling iterations. The calibration curves for both the training and validation groups of the nomogram model were approximately straight lines with a slope of 1. The Hosmer-Lemeshow goodness-of-fit test also demonstrated that the nomogram model fitting was good in both the groups ($\chi_{t}^{2}$ = 4.976, *p* = 0.760; $\chi_{v}^{2}$ = 7.236, *p* = 0.511). Decision Curve Analysis (DCA) results demonstrated high net benefit and clinical application value for the nomogram predictive model (Figures 4–6).

## 4 Discussion

This study utilized the CLHLS database to examine the prevalence of depression among older adult Chinese individuals with heart disease, revealing an association between the two. A predictive model of depression was constructed and validated using a training cohort, and a nomogram was developed to assess the risk of depression in these patients. The nomogram calculates a total score by summing the scores of various risk factors. This score, in conjunction with its relationship to the probability of depression occurrence, allows for the prediction of outcomes. By transforming complex regression equations into intuitive visual graphics, the nomogram offers a user-friendly approach for interpreting and assessing patient conditions.

### 4.1 Higher level of education is associated with lower risk of depression

Individuals with no formal education were found to be at a significantly higher risk of depression compared to those with at least an elementary or secondary education. This difference is likely due to the enhanced cognitive processing, problem-solving abilities, and broader social support networks typically associated with higher educational levels. Previous studies have also reported that older adult subjects with higher education levels show lower risk of depression than those with lower education levels (32, 33). This may be attributed to the better psychological adaptability and resource acquisition capabilities of patients with higher education (34–36). Furthermore, education facilitated cognitive stimulation and social engagement, which play a crucial role in alleviating the psychological burden associated with chronic diseases. Patients with higher education levels may be more likely to access health-related information and demonstrate proactive health management behaviors (37). Education is an important factor associated with psychological health, but its effect on depression has not yielded consistent results (38). Nikkheslat et al. reported that education level was not associated with the incidence of depression in older adult patients with heart disease (39).

### 4.2 Higher life satisfaction is associated with lower risk of depression

Older adult heart disease patients with low or moderate life satisfaction showed a higher risk of depression compared to those with higher life satisfaction. This was consistent with previous studies, which reported that lower life satisfaction of the individuals because of their current status resulted in a depressive mood (37, 40). Our findings also indicated that the subjective wellbeing and overall life satisfaction of the older adult heart disease patients was an effective management strategy for treating depression symptoms. This also emphasized that the quality of life can be improved through interventions targeting psychological health.

### 4.3 Better sleep quality is associated with lower risk of depression

The quality of sleep typically refers to the assessment of sleep duration, continuity, and depth. Several studies have indicated that both excessively long or short sleep durations impair cognitive function (41–43). The changes in cognitive function reduce the ability to integrate information and adversely affect emotional recognition and experiences (44). During this process, negative emotions are triggered and potentially lead to depressive symptoms. Sleep quality is one of the significant factors affecting the health of the older adults (45). Insomnia is a common sleep disorder reported by older adult individuals with depression. Several studies have reported that improving sleep quality by overcoming insomnia can reduce the risk of depression in the older adults (46). This is because poor sleep quality negatively affects the emotional regulation abilities of an individual and leads to low mood and depressive emotions. Sleep disturbances are associated with a variety of mental health issues, and good sleep quality is essential for maintaining physical and mental health. Therefore, improving sleep quality alleviates depressive symptoms (47). Poor sleep quality is a significant predictor of depression. Therefore, addressing sleep disturbances through behavioral interventions or pharmacological treatments is an important strategy for improving the psychological health outcomes of older adult patients with heart disease.

### 4.4 Frequency of watching TV is associated with comorbid depression in older adult patients with heart disease

This study also demonstrated a significant correlation between television watching frequency and the risk of depressive symptoms in the study subjects. The risk of depression was significantly higher in those patients who watched TV less frequently than those who watched television almost daily. Compared to individuals who watched television almost every day, the risk of depression was 2.413-fold higher for individuals who watched television at least once a week. The risk of depression increased to 2.734-fold for those who watched television at least and increased to 2.089-fold for those who abstained from watching television. These findings suggested that reduced television viewing was associated with depressive moods in the older adult cardiac disease patients.

Television is an important medium for information, entertainment, and relaxation, and can mitigate the onset of adverse emotional states (48). Furthermore, television viewing can enhance life quality and alleviate feelings of solitude among the older adults through engaging discussions about recent news or television narratives with peers (49). Our findings are partially consistent with the results of a study by Lin et al. (50), which reported that middle-aged and older adult individuals who did not watch television or engage in reading were more susceptible to depressive symptoms. Consequently, decreased television viewing may signify a loss of social support for the older adult cardiac disease patients and exacerbate their loneliness and depressive feelings.

However, in this study, patients who occasionally watched TV did not show a significant increase in the risk of depression. This may be related to factors such as individual differences, choice of viewing content, and viewing habits. Our data suggested that watching TV daily was a protective factor against depression in the older adult heart disease patients, but did not require prolonged TV watching. Some studies have reported that excessive TV watching may increase anxiety and depressive emotions of an individual (51). Therefore, the relationship between TV watching and mental health may be nonlinear and requires further research to determine the complex underlying mechanisms.

### 4.5 History of arthritis is associated with the incidence of comorbid depression in older adult patients with heart disease

Arthritis is a leading cause of chronic pain and functional impairment, significantly affecting quality of life. It is one of the most common sources of severe chronic pain and disability worldwide (52). Chronic pain from arthritis negatively impacts individuals' emotional wellbeing, coping mechanisms, and increases the risk of depression (53). In the older adult population, depression often manifests not only as emotional symptoms but also as fatigue, pain, and cognitive decline, leading to underdiagnosis in conditions such as osteoarthritis (54). Arthritis, with its accompanying pain, stiffness, and mobility restrictions, further diminishes quality of life (55). A meta-analysis conducted in China revealed a depression rate of up to 44.7% among patients with rheumatoid arthritis (56). Additionally, osteoarthritis patients face a depression risk twice that of the general population (57). The coexistence of arthritis and heart disease complicates health management, presenting both physical and psychological challenges. A history of arthritis was linked to an increased risk of depression in older adult heart disease patients. Conversely, those without a history of arthritis were less likely to develop depression. This may be attributed to the absence of chronic pain and physical limitations, which could enhance daily functioning and reduce reliance on medical resources, thereby alleviating both economic and psychological burden.

The relationship between a history of arthritis and depression may be influenced by various factors such as individual differences, disease severity, treatment methods, and psychological adaptability of the patients. Therefore, future studies are necessary to further identify the potential moderating variables that influence the specific relationship between a history of arthritis and the risk of depression in the older adult heart disease patients.

### 4.6 Limitations

Although the sample used in this study is considered representative, several limitations must be acknowledged. (1) The heart disease patients included varied in type and severity, which significantly affects depression levels. However, the database did not provide detailed information on the specific types of heart disease. (2) The study's variables were limited, and relevant laboratory tests for the older adult heart disease population could provide more insight, requiring further validation. (3) The depression scoring scale relied on self-reported data, which may introduce recall bias. (4) The CLHLS database represents only the older adult Chinese population, and its applicability to other regions warrants further investigation.

## 5 Conclusion

The incidence of comorbid depression in older adult heart disease patients is considerable and deserves greater attention. This study identified several factors—education level, life satisfaction, arthritis history, television viewing time, and sleep quality—that influence the likelihood of comorbid depression in this population. A nomogram was developed to predict depression risk, and after evaluating the model's discrimination, calibration, and clinical applicability, we conclude that it can effectively identify at-risk patients in medical practice, offering significant clinical value.

## Data Availability

The original contributions presented in the study are included in the article/supplementary material, further inquiries can be directed to the corresponding author.
